# Review on a cesarean section in the cow: Its incision approaches, relative advantage, and disadvantages

**DOI:** 10.1002/vms3.808

**Published:** 2022-04-27

**Authors:** Solomon Amente Adugna, Jiregna Dugassa Kitessa, Cheru Telila Feyissa, Sultan Aliyi Adem

**Affiliations:** ^1^ Department of Veterinary Medicine College of Veterinary Medicine and Clinical Science Wollega University Nekemte Ethiopia; ^2^ Department of Clinical Studies College of Veterinary Medicine and Agriculture Addis Ababa University Bishoftu Ethiopia; ^3^ Department of Veterinary Teaching Hospital College of Veterinary Medicine and Agriculture Addis Ababa University Bishoftu Ethiopia; ^4^ Department of Animal Science College of Agriculture and Natural Resources Madda Walabu University Robe Ethiopia

**Keywords:** cesarean section, cow, dystocia, surgical approach

## Abstract

Dystocia is an abnormal and difficult birth in which the first or the second stage of labour is markedly prolonged and subsequently found impossible for the dam to deliver without artificial aid. In cattle, it can be relieved by different obstetric methods, including the cesarean operation and fetotomy. Caesarean section is the extraction of the fetus or foeti from the dam, through a surgical opening in the abdominal wall and the uterus. This surgical method can be performed by about eight alternative surgical approaches in bovines with its advantages and disadvantages. However, the selection is dependent on many factors like the type of dystocia, the cows and environmental conditions, the availability of assistants, and the surgeon's preference. For cows, most surgeons use a standing left paralumbar celiotomy. However, the left oblique approach is also preferable under most circumstances because the uterus is readily exteriorized, limiting peritoneal cavity contamination. Besides, alternative approaches are also available that will further limit the potential for contamination but many junior surgeons perform the left paralumbar celiotomy using the same approach each time due to their comfort with one specific approach or lack of familiarity with other available options. Therefore, the objective of this review is to provide basic insights and highlight the cesarean section incision approaches with their relative advantages and disadvantages in cows.

## Graphical abstract

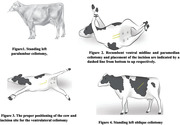

## 1 BACKGROUND

Dystocia is defined as difficulty in parturition as opposed to normal parturition and is characterized by prolonged first or second labour (Dhindsa et al., 2019). It is one of the major problems in the dairy industry that increases cow and calf mortality, decreases milk yield, delays uterine involution, and reproductive performance, ultimately resulting in substantial financial loss (Yohannes et al., 2018). Among all domestic animals, cattle and buffalo are considered to be the most suspicious species having the highest incidence rate of dystocia (Hasan et al., 2017). In cattle, it can be relieved by different obstetric methods, including the cesarean operation and fetotomy (Ajeel, 2019). The caesarean section involves the extraction of the fetus or foeti from the dam, through a surgical opening in the abdominal wall and the uterus. It is commonly indicated in cases of dystocia when a calf cannot be delivered by a normal parturition cascade (Fesseha et al., 2020).

The cesarean section dilemma has been based on poor dam survival rates and poor fertility; however, many reports depicted that dam survival is high when the operation is performed early. This is mainly due to the development of peritonitis in delayed cases and also possibly following cesarean section due to leakage of uterine fluids, rupture of suture material, and knot failure. Consequently, peritonitis will lead to uterine adhesions that invariably result in death or infertility (Dhindsa et al., 2019). Regardless of its disadvantages, the purpose of the cesarean section is to save the lives of the cow and/or calf. However, the successfulness of this surgical treatment may also be affected by several factors such as the nature of the cow, asepsis, surgical technique and approaches, calf viability, and the nature of the uterus (Newman & Anderson, 2005).

Moreover, a good surgical technique, such as gentle tissue handling, selection of appropriate suture materials and patterns, and adequate folding of the uterine incision to prevent leakage, combined with antibiotics and anti‐inflammatory medication when indicated, can help to minimize detrimental adhesions and possible post‐operative complications that may adversely affect the future reproductive efficiency of the cow (Fesseha et al., 2020). There are different surgical approaches for cesarean section in cows with variable degrees of advantages and disadvantages. These include standing left and right paralumbar celiotomy, recumbent left and right paralumbar celiotomy, recumbent ventral midline celiotomy, ventral paramedian celiotomy, ventrolateral celiotomy, and standing left oblique celiotomy (Schultz et al., 2008). The selection of an appropriate approach depends on the type of dystocia, health status of the cow, environmental conditions, availability of assistants, and the surgeon's preference (Schultz et al., 2008).

The selected surgical approach in turn can also determine anaesthetic application technique. The most common techniques are the proximal paravertebral and distal paravertebral, inverted ‘L’, and line blocks (Ajeel, 2019). Besides, animal control and anaesthesia also depend on the type of breed and nature of the cow, available space, veterinarian training, experience, and confidence, whether to do a cesarean section on standing or a recumbent. The operation can be performed using sedation and tying the legs forward and more appropriate in cases where no chute is present and if the cow may not remain standing for the duration of the surgery (Newman & Anderson, 2005).

Even though there are different and alternative surgical approaches for cesarean section in the cow, most surgeons use a standing left paralumbar celiotomy. However, the left oblique approach is preferable under most circumstances because the uterus is readily exteriorized, limiting peritoneal cavity contamination. Most of the time, many junior surgeons also perform left paralumbar celiotomy which may be due to their comfort with one specific approach or lack of familiarity with other available options (Schultz et al., 2008). Furthermore, the selection of preferable alternative surgical approaches and techniques also greatly influences the outcome of a cesarean section. Even though there are different findings of cesarean sections in various female animals in the form of case reports and studies, the facts on the selection of incision approaches, their pros and cons are not well documented and commercialized for further frame of reference and guideline, especially in cows. Therefore, the objectives of this review are given below.

- To explain the advantages, disadvantages, and indications for each of the different cesarean section approaches in cows.
- To provide veterinarians with basic insights concerning the selection of cesarean section incision approaches in cows.

## 2 OVERVIEW OF CESAREAN SECTION IN COW

Even though dystocia is one of the emergency cases, different factors should have to be assessed before performing surgery for optimum success in cows. These may include the skill and speed of the surgeon, duration of dystocia, the physical condition of the dam, surgical environment, concurrent disease, and nature of the calf (Ajeel, 2019). Ideally, it is carried out when a live calf cannot be delivered after 15–20 min of manipulation. The need for urgent intervention is indicated if there is evidence of fetal hypoxia, as shown by hyperactive movements of the fetus and expulsion of the meconium, identifiable in the amniotic fluid (Weldeyohanes, 2020). Furthermore, dystocia can be indicated for several reasons (Table 1).

**TABLE 1:** Indications for cesarean section

Maternal factors/dystocia	Fetal factors/dystocia
Irreducible uterine torsion	Fetal abnormalities (hydrocephalus, fetal ascites, anasarca, cleft palate)
Hydroallantois/hydroamnion	
Narrow pelvis/pelvic fracture	Fetal monsters
Incomplete cervical dilation	Fetal mal disposition
Extra‐uterine pregnancy	Fetal oversize/emphysema
Uterine inertia	Mummified fetus
Uterine rupture	Macerated fetus
Urinary bladder carcinoma	
Irreducible prolapse mass	
Bicornual pregnancy	

*Source*: Thangamani et al. (2018).

### 2.1 Approaches of cesarean section

#### 2.1.1 Standing left paralumbar laparotomy

**FIGURE 1:**
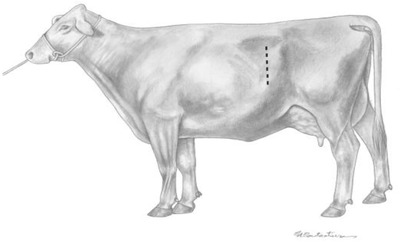
Standing left paralumbar celiotomy. *Source*: Schultz et al. (2008)

The standing left paralumbar laparotomy is the most commonly used approach for uncomplicated dystocia. The incision is made vertically in the middle of the paralumbar fossa, starting approximately 10 cm ventral to the transverse processes of the lumbar vertebrae and continuing ventrally (Figure 1), far enough to allow removal of the calf. Closure of the abdominal wall is straightforward and relatively easy. An absorbable suture is used to close the abdominal musculature (Schultz et al., 2008).

The primary advantage of this approach is that the rumen prevents evisceration of the small intestines, but rumen prolapse may occur if straining during surgery (Newman & Anderson, 2005). However, the disadvantage of this procedure includes an inability of the cow to stand throughout the procedure and large fetuses that preclude exteriorization of the uterus. Thus, lifting a uterus and calf to the paralumbar incision is usually difficult and occasionally impossible for some practitioners (Schultz et al., 2008).

#### 2.1.2 Standing right paralumbar laparotomy

The most important difference between the left and right paralumbar approaches is the difficulty in keeping intestines in the peritoneal cavity with the right paralumbar approach (Newman & Anderson, 2005). This approach is relatively preferable when a large calf can be palpated in the right horn with its limbs directed towards the right side of the cow, a lot of scar tissue/adhesions on the left side in a cow that has had repeated caesareans and on cows with the hydrotic condition of the uterus (Newman, 2008). In the case of an animal with the hydrotic condition, the location of the rumen and the increased size of the uterus seem to force the uterus into the right paralumbar fossa, permitting easier removal of the fetus, limiting abdominal contamination, and permitting the surgeon to leave substantial volumes of fluid within the lumen of the uterus (Schultz et al., 2008).

#### 2.1.3 Recumbent left paralumbar laparotomy

This approach differs little from the standing left paralumbar approach in that additional assistance is nearly always needed to cast the cow, if not recumbent already, and to place the cow in right lateral recumbency, and the incision is made slightly more ventral than in the standing left paralumbar laparotomy (Jos, 2020). However, exteriorization of the uterus is often difficult because the gravid uterus falls away from the incision. The closure is more difficult than when the standing left paralumbar approach is used, due to increased tension on the muscle layers, but it is rarely problematic (Schultz et al., 2008).

#### 2.1.4 Recumbent right paralumbar celiotomy

This approach is very seldom used, as it is very similar to that of recumbent left paralumbar celiotomy and has the additional complication of not having the rumen to retain the abdominal viscera (Schultz et al., 2008).

#### 2.1.5 Recumbent ventral midline celiotomy

**FIGURE 2:**
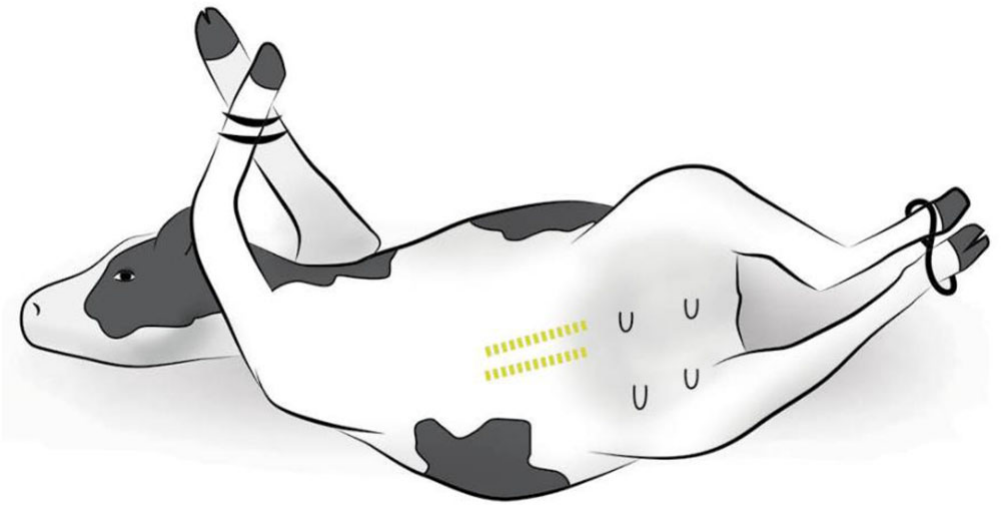
Recumbent ventral midline and paramedian celiotomy and placement of the incision are indicated by a dashed line from bottom to up, respectively. *Source*: Schultz et al. (2008)

This incision approach is performed starting 5–7 cm caudal to the umbilicus and extended as caudal as required on body wall layers after positioning the cow in dorsal recumbence, leaning toward the surgeon at a 45‐degree angle (Figure 2). The structures to be incised are skin, subcutis, and the linea alba (Schultz et al., 2008). Once the peritoneal cavity has been opened, it may be necessary to pull the greater omentum cranially to expose the uterus. Exteriorizing the uterus is facilitated by untying the hind feet only and temporarily laying the hind limbs flat on the ground. After removal of the fetus and closure of the uterus, the cow is repositioned in dorsal recumbency and the linea alba is closed; however, the closure of the abdominal wall is often difficult.

The authors typically close the linea alba with polyglactin 910 (#2 Vicryl) in an everting interrupted horizontal mattress pattern may be due to the presence of tension. The integrity of abdominal wall closure is critical and negligent closure may result in either abdominal wall herniation or, in severe cases, evisceration of the abdominal organs (Sexton, 1954). The approach is relatively preferable for most surgeons when there is an emphysematous fetus (Schultz et al., 2008). The disadvantage of this approach is its limited use in the older cows having large udder and dairy cows due to the presence of increased ventral vasculature. Besides, the midline approach likely requires the longest incision due to the inflexible nature of the linea alba. Therefore, this approach should not be used when a large incision may deem necessary (Newman & Anderson, 2005).

#### 2.1.6 Recumbent ventral paramedian celiotomy

The pros and cons as well as its control are more or less similar to the midline approach with few differences. In this approach, the cow can be sedated and put in dorsal recumbency leaning 45° towards the surgeon with both feet tied. The abdominal wall incision is placed parallel and approximately 5 cm lateral to the linea alba and medial to the milk vein (Figure 2). Some authors have postulated that the abdominal wall closure of the paramedian approach is more secure than that of the ventral midline approach. But neither the internal sheath of the rectus abdominis muscle nor the rectus abdominis muscle has substantial holding properties. It would seem that a one‐layer abdominal closure is easier and, hence, preferable to a three‐layer. Although both ventral midline and paramedian methods offer good access and exteriorization of the uterus and could be used for emphysematous calf removal, they are problematic for several reasons: heavy sedation and manpower are required to put and keep the cow in dorsal recumbency; the abdominal incision can be difficult to close, and there is a considerable risk of abdominal wall herniation or evisceration if abdominal wall closure is poor. Not only these but also the probability of wound infection is more of an issue due to its location on the ventral part of the abdominal wall (Schultz et al., 2008).

#### 2.1.7 Ventrolateral celiotomy

**FIGURE 3:**
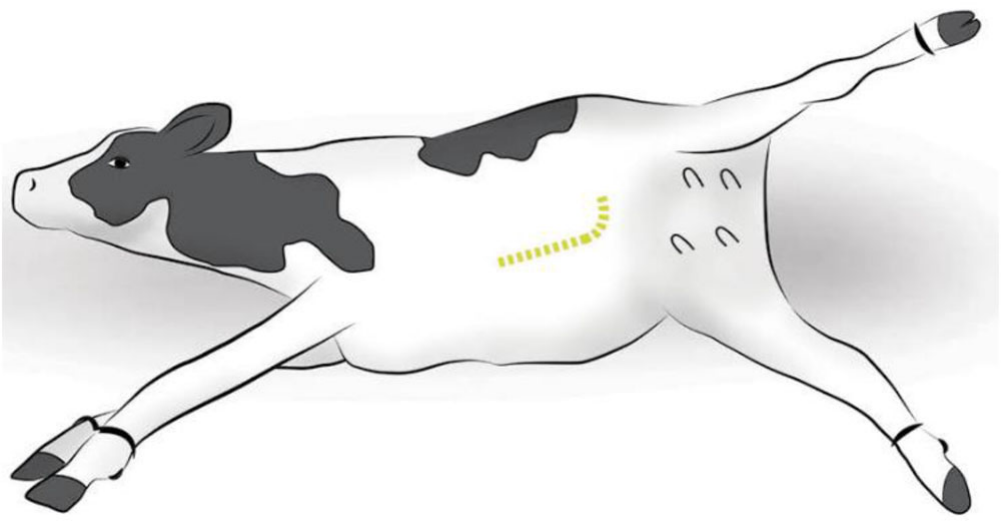
The proper positioning of the cow and incision site for the ventrolateral celiotomy. The placement of the incision is indicated by the dashed line. *Source*: Schultz et al. (2008)

This incision approach is suited better for older beef cows or dairy cows (Newman & Anderson, 2005). The cow is positioned in right lateral recumbency and involves the extension of the hind limbs caudally and abduction of the upper limb for the best exposure to the incision site (Figure 3). A curved incision is made starting 20 cm dorsal to the attachment of udder, medial to the fold of the stifle, and it is continued about 50 cm cranio‐ventrally to allow exteriorization of the gravid horn. Once exteriorized, the gravid horn is incised along its ventral aspect, the calf is removed, and the uterus is sutured using absorbable suture material in one or two layers by modified Cushing which is also known as the Utrecht method. The muscles are closed in three layers with six to eight metric absorbable suture materials in a continuous pattern (Schultz et al., 2008).

**FIGURE 4:**
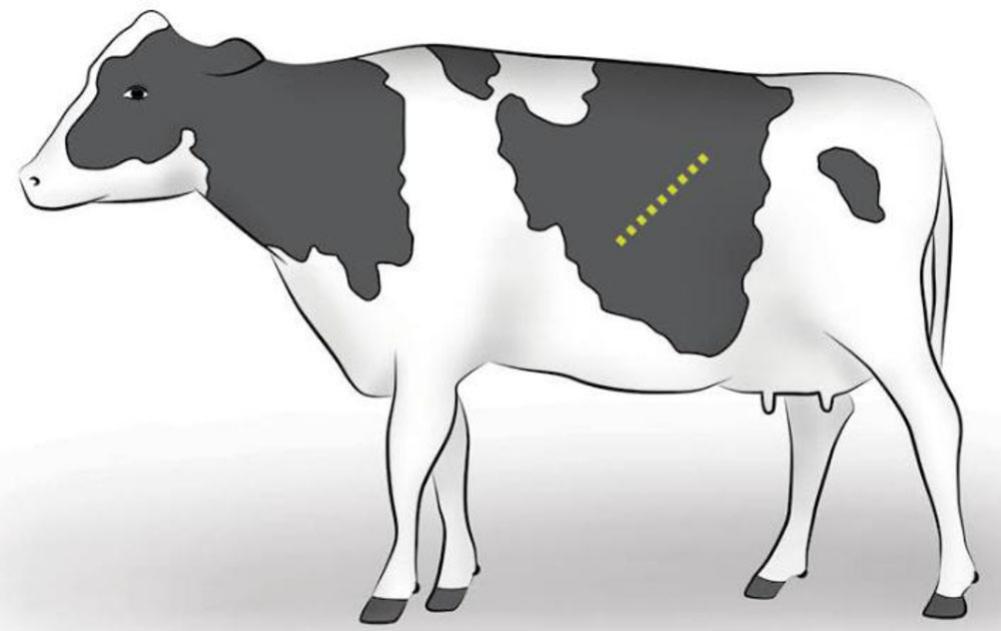
Standing left oblique celiotomy. *Source*: Schultz et al. (2008)

This approach is a novel way of permitting exteriorization of the uterus, making it suitable for easy removal of a large emphysematous fetus. In a cow with a large udder, the incision is more readily extended caudally than the ventral midline or ventral paramedian approach. Besides, this incision is not readily visible in a standing cow and offers additional advantages especially for the cows going to be sold soon following the operation, avoids the well‐vascularized musculature of the flank, does not require dorsal recumbency, and allows excellent exteriorization of the uterus. However, its disadvantages are difficulty during the closure of the incision as more tension is placed on the muscle layers predisposing for the likely occurrence of surgical site infection, and the presence of less secured abdominal wall closure relative to the ventral midline and ventral paramedian approaches and therefore, more prone to herniation and evisceration of the cow (Schultz et al., 2008).

#### 2.1.8 Standing and recumbent left oblique celiotomy

More recently, a left oblique flank approach in standing cows is performed (Figure 4). The incision starts 4–6 cm ventral and cranial to the tuber coxae and is extended cranioventrally at a 45°‐angle toward the caudal rib where it stops. The approach offers a few advantages such as relative accessibility of the uterine horn apex, facilitating manipulation and exteriorization of the uterus, and can be particularly useful if there is a very heavy calf present as it requires less physical strength to exteriorize the calf. It does not require much more help than what is required for a standard standing‐left approach and there is much less risk of herniation or evisceration. Besides, the internal abdominal oblique muscle is incised parallel to the muscle fibres; the abdominal viscera apply tension to this muscle, facilitating apposition during closure. However, the incision is larger and extends more cranio‐ventrally compared with the classic vertical flank incision (Newman & Anderson, 2005).

This approach allows easier access to the uterus and can also be used in a recumbent approach with the incision starting lower. The recumbent approach is more appropriate than the standing method for the emphysematous fetus as it results in less abdominal contamination and better exteriorization of the uterus. This approach holds also distinct advantages for surgeons with either smaller stature or less physical strength. The patient must be adequately restrained and must be able to remain to stand, but as with the other standing procedures, minimal assistance is needed (Schultz et al., 2008).

## 3 CONCLUSION AND RECOMMENDATIONS

Cesarean section is one of the emergency surgical interventions performed for the delivery of calves during dystocia by opening the abdominal cavity. It may be performed due to several reasons which emanate from maternal and/or fetal factors. The procedure requires restraint which in turn relies on the breed, space, light, availability of assistance, location, and the veterinarian's training, experience, and confidence. There are several surgical approaches for cesarean section in a cow with each its relative advantage and disadvantages. However, most surgeons use a standing left paralumbar laparotomy. But the left oblique approach is preferable under most circumstances because the uterus is readily exteriorized, limiting peritoneal cavity contamination. Furthermore, the selection of alternative approaches depends on various factors for the positive outcome of the procedure.

Based on the above conclusion, the following points were recommended:

- Veterinarians should have to consider alternate surgical approaches during cesarean section depending on the circumstances for a better outcome of the surgical procedure.
- Veterinarians should analyze the survival of the cow and the calf as well as maintenance of post‐operative productivity before selecting a particular approach.

## CONFLICT OF INTEREST

The authors declare no conflict of interest.

## ETHICS STATEMENT

The authors confirm that the ethical policies of the journal, as noted on the journal's author guidelines page, have been adhered to. No ethical approval was required as this is a review article with no original research data.

## AUTHOR CONTRIBUTIONS

All authors contributed to the manuscript to final submission. Conceptualization and writing the original draft were performed by Solomon Amente Adugna, and validation and supervision were majorly done by Jiregna Dugassa Kitessa, while reviewing and editing were done by Cheru Telila Feyissa and Sultan Aliyi Adem. Finally, all authors read and approved the final manuscript submission.

### PEER REVIEW

The peer review history for this article is available at https://publons.com/publon/10.1002/vms3.808

## Data Availability

NA.
